# Genetic and environmental architecture of conscientiousness in adolescence

**DOI:** 10.1038/s41598-021-82781-5

**Published:** 2021-02-05

**Authors:** Yusuke Takahashi, Anqing Zheng, Shinji Yamagata, Juko Ando

**Affiliations:** 1 Graduate School of Education, Kyoto University, Yoshida Honmachi, Sakyo-ku, Kyoto, 6068501 Japan; 2 Department of Psychology, University of Illinois at Urbana-Champaign, Champaign, USA; 3 Graduate School of Education and Human Development, Nagoya University, Nagoya, Japan; 4 Faculty of Letters, Keio University, Tokyo, Japan

**Keywords:** Behavioural genetics, Human behaviour

## Abstract

Using a genetically informative design (about 2000 twin pairs), we investigated the phenotypic and genetic and environmental architecture of a broad construct of conscientiousness (including conscientiousness per se, effortful control, self-control, and grit). These four different measures were substantially correlated; the coefficients ranged from 0.74 (0.72–0.76) to 0.79 (0.76–0.80). Univariate genetic analyses revealed that individual differences in conscientiousness measures were moderately attributable to additive genetic factors, to an extent ranging from 62 (58–65) to 64% (61–67%); we obtained no evidence that shared environmental influences were observed. Multivariate genetic analyses showed that for the four measures used to assess conscientiousness, genetic correlations were stronger than the corresponding non-shared environmental correlations, and that a latent common factor accounted for over 84% of the genetic variance. Our findings suggest that individual differences in the four measures of conscientiousness are not distinguishable at both the phenotypic and behavioural genetic levels, and that the overlap was substantially attributable to genetic factors.

## Introduction

Individuals differ in a variety of psychological features, such as socio-emotional skills, that affect how they move through the educational system. Socio-emotional skills reflect personality traits, non-cognitive ability, twenty-first century skills, soft skills, and character strength^1^. In recent years, these concepts have attracted increasing attention in the fields of education, economics and health care^2,3^. Grit, in particular, is a useful construct explaining for why some individuals perform better than others even after controlling for cognitive abilities (i.e. intelligence)^4^. Although immense efforts have been devoted to establishing novel measures of specific psychological constructs, criticisms include the “jangle fallacy,” a term first coined by Kelley^5^ to describe how different measures with apparently dissimilar labels might measure similar constructs; thus, the supposedly related traits may have a common core^6^. Thus, it is important to evaluate newly developed measures using widely accepted measurement systems, such as the Big Five^7^, to explore whether the new constructs indeed add unique theoretical information, and whether the constructs are informative when developing policy.

Grit is defined as perseverance and passion in those with long-term goals, strenuous efforts to address challenges, and maintenance of effort and interest for years despite failure, adversity, and plateaus in progress. Of the various supposed socio-emotional skills, grit has emerged as a significant predictor of life success and educational achievement^8^. Grit has been incorporated into educational policies, with the aim of children developing “non-cognitive” skills^9^, although the construct validity of grit and its relationship to other constructs has not really been addressed.

However, by definition, grit is arguably similar to a relatively narrow aspect of conscientiousness and is derived using a global construct involving the Big Five: self-controlled, responsible to others, hardworking, orderly behaviour, and a rule-abiding nature^10^. One recent meta-analysis found that the grit scale was only marginally more useful than pooled conscientiousness measures^11^. A second study found that grit and conscientiousness were highly correlated when the estimates were greater than 0.5^12^. The relationship between grit and other aspects of conscientiousness (such as self-control) has been explored^13^. Although Duckworth and Gross^14^ discussed both grit and self-control, the capacity to change one’s responses, bringing them into line with the ideals, values, morals, and social expectations of others, and using them to pursue long-term goals^15^, are related but distinct determinants of success, and are usually treated as conceptually interchangeable^10^. Likewise, effortful control (or regulation of effort) and the ability to inhibit prepotent responses^16^ have been considered to be developmental predecessors of conscientiousness^17^. In college students, Muenks et al.^13^ found that grit correlated highly with both of these constructs, with coefficients of 0.67 for self-control and 0.41 for effortful control. Together, these data suggest that although slight differences are apparent, similar psychological constructs have been defined by different names within a segmented professional discipline. Given such commonalities among them, it seems likely that a common factor may underlie several aspects of conscientiousness, conscientiousness per se from personality psychology, effortful control from developmental psychology, self-control from social psychology, and grit from positive psychology. However, no prior study has explored whether a single common factor captures the interrelationships among the four constructs.

Given the potential impact of grit on educational policy in advanced countries such as the United States and the United Kingdom^9^, it is essential to understand the relationship between this trait and other similar constructs. It is also important to examine the genetic and environmental structure of various constructs, including grit, to inform policy and interventions effectively. As Duckworth commented, “Science says grit comes from both nature and nurture^18^”; it is thus essential to explore whether the construct validity of the grit concept occurs through a genetic or environmental pathway. In this respect, behavioural genetic approaches such as twin studies can be most useful. Apart from exploring correlations between measures of observed phenotypes, a twin design which uses the differences between monozygotic (MZ) and dizygotic (DZ) twin siblings allow estimation of how variations in the observed results are associated with genetic differences. Using a behavioural genetic design, we can investigate not only how measures are affected by genetic differences but also the genetic and environmental architectures of the associations among measures^19–21^.

Few studies have used genetically informative designs to explore the genetic and environmental influence of grit or any associations of grit with other related concepts^12,22,23^; even less is known about the genetic and environmental architecture of conscientiousness in young subjects. Tucker-Drob et al.^24^ found that grit was 48% heritable, and that about 50% of the genetic variance was shared with that of conscientiousness. However, to date, no effort has been made to explore the genetic and environmental associations among various measures of conscientiousness when assessing individual differences within a broader conscientiousness construct (i.e. conscientiousness per se, effortful control, self-control, and grit), or whether there is a common dimension accounting for their overlap.

Therefore, we addressed the following three core questions regarding the phenotypic and genetic and environmental relationships between conscientiousness, effortful control, self-control, and grit. First, how are these four measures associated at the phenotypic level? We expected that substantial correlations would be evident, given their conceptual similarities. Second, to what extent are such measures heritable? We estimated heritabilities, and the genetic and environmental origins of individual differences in the four conscientiousness-related measures, using a large Japanese adolescent twin sample (about 2000 pairs). Third, are the constructs genetically coherent, or do they exhibit independent genetic and environmental dimensions? In other words, if it is assumed that several psychological constructs are mutually phenotypically correlated, the coherence (or incoherence) of the genetic and environmental architectures should be empirically investigated to determine the source(s) of covariance. We explored the extent to which the four conscientiousness-related measures were attributable to common genetic and environmental factors, to test, in particular, whether grit might be associated with unique factors. If grit, which has attracted widespread enthusiasm, is indeed a novel independent determinant of life outcomes, grit should be genetically and/or environmentally independent of any broader construct of conscientiousness. Alternatively, if grit is essentially one component of the broad construct of conscientiousness, genetic and environmental influences should be largely attributable to a common factor, with a little unique genetic or environmental variance. Based on our interpretations of the results of previous studies, we predicted that the phenotypic correlations among the four conscientiousness measures would be primarily genetic, and that their architecture would be genetically and environmentally coherent.

## Results

### Phenotypic analyses of conscientiousness measures

Table 1 presents the means and standard deviations of individual measures, phenotypic correlations among conscientiousness-related measures, and partial correlations after controlling for twin age and sex, and the intraclass twin correlations of each phenotype. As expected, the phenotypic correlations were substantially correlated; even after controlling for twin age and sex, the partial correlations remained substantially correlated. Thus, all subsequent analyses (including behavioural genetic modelling) were conducted based on scores controlled for age and sex effects. The correlations between the original scores and the residuals were all above 0.989, indicating that age and sex had limited influence on conscientiousness-related scales.

**Table 1:** Descriptive statistics, and phenotypic and intraclass twin correlations (with 95% confidence intervals) of the four conscientiousness variables.

		*M*	*SD*	Phenotypic correlations [with 95% CI]				Intraclass correlations [with 95% CI]	
				1	2	3	4	MZ	DZ
1	Conscientiousness	3.13	0.41	–	0.78	0.79	0.78	0.61	0.35
					[0.76, 0.80]	[0.77, 0.80]	[0.76, 0.80]	[0.57, 0.64]	[0.28, 0.42]
2	Effortful control	3.15	0.52	0.79	–	0.77	0.75	0.56	0.31
				[0.77, 0.80]		[0.75, 0.79]	[0.73, 0.77]	[0.52, 0.60]	[0.24, 0.38]
3	Self-control	3.13	0.47	0.79	0.77	–	0.74	0.58	0.32
				[0.78, 0.81]	[0.75, 0.79]		[0.72, 0.76]	[0.55, 0.62]	[0.25, 0.39]
4	Grit	3.14	0.42	0.79	0.75	0.74	–	0.58	0.35
				[0.76, 0.80]	[0.73, 0.77]	[0.72, 0.76]		[0.54, 0.61]	[0.28, 0.42]

In the phenotypic correlation matrix, numbers below the diagonal are simple correlations, and those above the diagonal partial correlations after controlling for age and sex.

In the phenotypic correlation matrix, numbers below the diagonal are simple correlations, and those above the diagonal partial correlations after controlling for age and sex.

The grit score was highly (and positively) associated with the conscientiousness, effortful control, and self-control scores (*r* values with 95% confidence intervals [CIs] 0.79 [0.76, 0.80]; 0.75 [0.73, 0.77]; and 0.74 [0.72, 0.76], respectively). Additionally, the MZ twin correlations were higher than the DZ twin correlations for all four variables, indicating genetic influences. We next performed confirmatory factor analysis using the R package *lavaan*^25^. The single-factor model fitted the data reasonably well (CFI = 0.999, RMSEA = 0.045, and SRMR = 0.005), indicating that the four measures were phenotypically coherent.

### Univariate genetic analyses of conscientiousness measures

Given that the MZ correlations were less than twice those of the DZ correlations for all phenotypes, it was more likely that a shared environmental effect (rather than a non-additive genetic effect; Table 1) contributed to similarity within pairs of twins. We first fitted an ACE model to the raw data, and compared this to AE, CE, and E models. For all four conscientiousness measures, the fit indices of the AE models were noticeably smaller than those of other models based on the Bayesian Information Criterion (BIC, Table 2)^26^. Overall, univariate genetic analyses revealed that all four conscientiousness phenotypes were moderately heritable; the coefficients ranged from 0.62 (0.58, 0.65) for grit to 0.64 (0.61, 0.67) for conscientiousness in the absence of any common environmental influence.

**Table 2:** Univariate model fitting results of the four conscientiousness variables with parametric estimates (and 95% confidence intervals).

	A	C	E	*χ*^2^	*df*	Δ*χ*^2^	Δ*df*	*p*	AIC	BIC
**Conscientiousness**										
ACE	0.54	0.10	0.36	3447.57	3912	–	–	–	− 4376.43	− 26,204.14
	[0.42, 0.66]	[0.00, 0.21]	[0.33, 0.40]							
AE	0.64	–	0.36	3450.59	3913	3.02	1	0.08	− 4375.42	− 26,208.70
	[0.61, 0.67]		[0.33, 0.39]							
CE	–	0.51	0.49	3530.50	3913	82.93	1	< 0.01	− 4295.50	− 26,128.79
		[0.48, 0.54]	[0.46, 0.52]							
E	–	–	1.00	4117.54	3914	669.97	2	< 0.01	− 3710.46	− 25,549.33
**Effortful control**										
ACE	0.63	0.00	0.37	5175.97	3912	–	–	–	− 2648.03	− 24,475.73
	[0.55, 0.66]	[0.00, 0.07]	[0.34, 0.41]							
AE	0.63	–	0.37	5175.97	3913	0.00	1	1.00	− 2650.03	− 24,483.31
	[0.59, 0.66]		[0.34, 0.41]							
CE	–	0.46	0.54	5295.21	3913	119.24	1	< 0.01	− 2530.79	− 24,364.08
		[0.42, 0.49]	[0.51, 0.58]							
E	–	–	1.00	5762.04	3914	586.07	2	< 0.01	− 2065.96	− 23,904.82
**Self-control**										
ACE	0.63	0.00	0.37	4605.65	3912	–	–	–	− 3218.35	− 25,046.05
	[0.53, 0.67]	[0.00, 0.10]	[0.34, 0.40]							
AE	0.63	–	0.37	4605.65	3913	0.00	1	1.00	− 3220.35	− 25,053.63
	[0.60, 0.67]		[0.34, 0.40]							
CE	–	0.48	0.52	4717.63	3913	111.98	1	< 0.01	− 3108.37	− 24,941.66
		[0.44, 0.51]	[0.49, 0.56]							
E	–	–	1.00	5219.20	3914	613.55	2	< 0.01	− 2608.80	− 24,447.66
**Grit**										
ACE	0.59	0.03	0.39	3810.32	3912	–	–	–	− 4013.68	− 25,841.38
	[0.46, 0.65]	[0.00, 0.14]	[0.35, 0.42]							
AE	0.62	–	0.38	3810.55	3913	0.23	1	0.63	− 4015.45	− 25,848.73
	[0.58, 0.65]		[0.35, 0.42]							
CE	–	0.48	0.52	3898.15	3913	87.83	1	< 0.01	− 3927.85	− 25,761.14
		[0.45, 0.52]	[0.48, 0.55]							
E	–	–	1.00	4417.09	3914	606.77	2	< 0.01	− 3410.91	− 25,249.77

### Multivariate genetic analyses of conscientiousness measures

As we improved model fit by constraining all shared environmental influences to zero, all subsequent multivariate genetic analyses were performed assuming that only A and E influenced the four phenotypes. The extent to which these factors contributed to the observed phenotypic correlations were calculated. Table 3 lists the genetic and non-shared environmental correlations estimated using the correlated-factors model; to examine the genetic and non-shared environmental correlations, we transformed the Cholesky decomposition solutions for all four phenotypes into correlated-factors model solutions because they are mathematically equivalent^27^. The genetic correlations (*r*_G_ values) were stronger than the non-shared environmental correlations (*r*_E_ values); the *r*_G_ values ranged from 0.82 (0.79, 0.84) to 0.91 (0.89, 0.93) and the *r*_E_ values from 0.56 (0.52, 0.59) to 0.62 (0.59, 0.65). A genetic (or non-shared environmental) correlation indicates the extent to which a genetic (or non-shared environmental) influence on one phenotype overlaps with those on other phenotypes, regardless of heritability (or environmentability).

**Table 3:** Genetic and non-shared environmental correlations among conscientiousness measures (with 95% confidence intervals).

	Conscientiousness	Effortful control	Self-control	Grit
Conscientiousness	–	0.58	0.62	0.60
		[0.54, 0.61]	[0.59, 0.65]	[0.57, 0.63]
Effortful control	0.91	–	0.59	0.56
	[0.89, 0.93]		[0.55, 0.62]	[0.52, 0.59]
Self-control	0.88	0.89	–	0.61
	[0.86, 0.90]	[0.87, 0.91]		[0.58, 0.64]
Grit	0.90	0.89	0.82	–
	[0.88, 0.91]	[0.86, 0.91]	[0.79, 0.84]	

Numbers below the diagonal indicate genetic correlations, and those above the diagonal non-shared environmental correlations.

**Figure 1:**
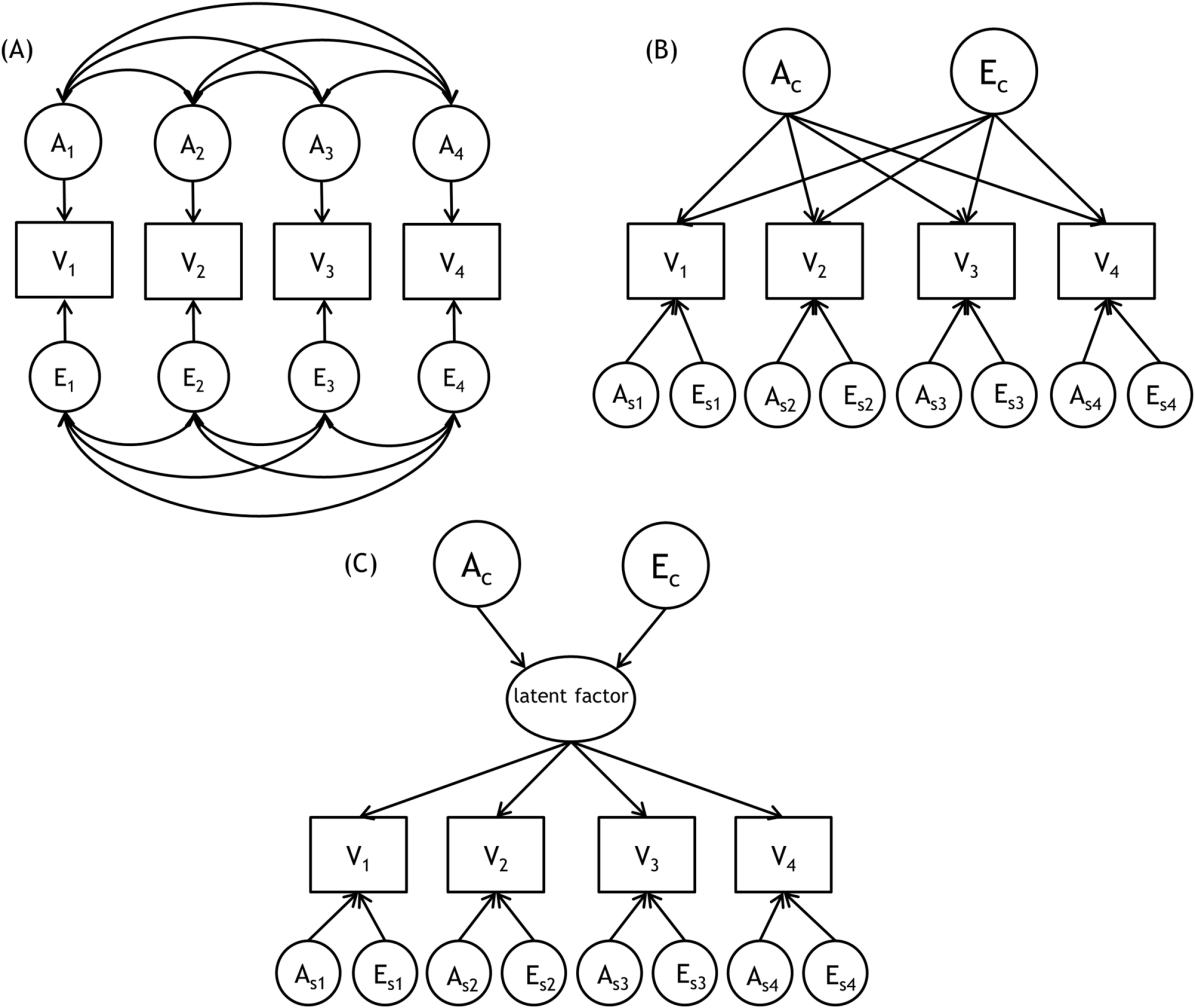
Path diagrams of multivariate genetic models: (**A**) correlated factors model (transformed from full Cholesky decomposition), (**B**) independent pathway model, and (**C**) Common pathway model, showing one twin only for simplicity.

**Figure 2:**
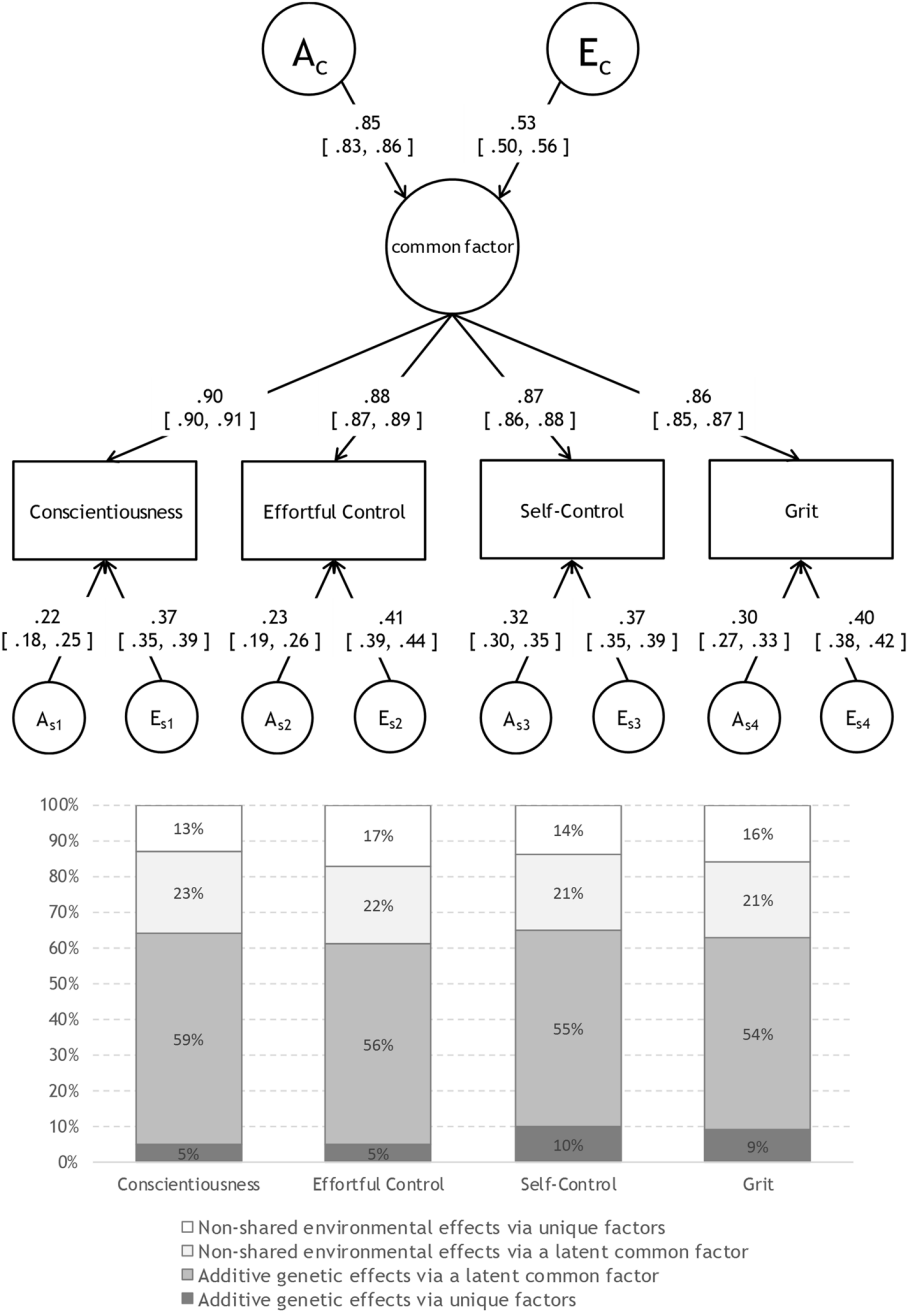
AE common pathway model for conscientiousness-related measures with standardised estimates (and 95% confidence intervals) alongside bar charts for the percent variance explained.

To establish the genetic and environmental architecture of the overlap, and the distinctive features of the four conscientiousness measures, we compared correlated-factors, independent pathway, and common pathway models (see the “Methods” section and Fig. 1). As shown in Table 4, the AE common pathway model (BIC =  − 113,095.26) shown in Fig. 2 fit the data better than either the AE correlated-factors model (BIC =  − 113,075.39) or AE independent pathway model (BIC =  − 113,077.87). The estimates of the common pathway model indicated that their unity was attributable to the influence of a common latent factor that was 71.95% heritable, suggesting that the four psychological constructs were moderately interrelated because of shared genetic and non-shared environmental influences; low-level, specific genetic influences (4.79–10.37%) and low-level, specific, non-shared environmental influences (13.37–17.11%) were weakly contributed to the variance. A common genetic factor explained most of the genetic variance in conscientiousness (92.48% [≈ (0.90^2^ × 0.85^2^)/(0.90^2^ × 0.85^2^ + 0.22^2^)] (the following figures were similarly calculated: effortful control (91.30%); self-control (84.07%); and grit (85.35%), whereas specific genetic factors accounted for small proportions of the genetic variance (7.52 to 15.93%). A common non-shared environmental factor explained over half of the non-shared environmental variance, ranging from 55.98% for effortful control to 63.19% for conscientiousness; non-shared environmental variance accounted for less than half of these proportions (36.81 to 44.02%). Thus, the phenotypic covariation between each pair of conscientiousness measures was attributable primarily to genetic influences.

**Table 4:** Fit statistics for the multivariate models.

Models	− 2*LL*	*df*	Δ*χ*^2^	Δ*df*	*p*	AIC	BIC
AE correlated factors models	5470.79	15,640	–	–	–	− 25,809.21	− 113,075.39
AE independent pathway models	5498.62	15,644	27.83	4	< 0.01	− 25,789.38	− 113,077.87
AE common pathway models	5503.97	15,647	33.18	7	< 0.01	− 25,790.03	− 113,095.26

## Discussion

In this study, we examined phenotypic, and genetic and environmental influences on interrelationships among four psychological constructs related to conscientiousness in adolescent Japanese twins. This is the first study to reveal the genetic and environmental architecture of conscientiousness-related constructs assessed using four different measures from four different psychological disciplines: conscientiousness items from the Chernyshenko Conscientiousness scale (short form)^28^ (personality psychology); effortful control items from the Early Adolescent Temperament Questionnaire Revised^29^ (developmental psychology); self-control items from the Brief Self-Control scale^30^ (social psychology); and grit items from the Grit scale^8^ (positive psychology).

We confirmed the phenotypic and behavioural genetic (i.e. genetic and environmental) relationships among individual differences in a broader construct of conscientiousness in several significant ways. First, in line with previous findings^10,11^, the four conscientiousness measures were strongly phenotypically related, and such relationships held even after controlling for demographic variables (Table 1). This finding indicates that although the four measures originate from different psychological disciplines, they overlap at the phenotypic level and are conceptually interchangeable unless they exhibit specific, incremental predictive validities when exploring certain outcomes.

Second, we found that all four conscientiousness measures were moderately heritable; additive genetic effects explained over half of the phenotypic variances, and a negligible shared environmental effect was found (Table 2). The heritability estimates are slightly higher than previous estimates for various personality traits but, basically, replicate the earlier findings; 30–60% of individual differences in conscientiousness are genetic in nature and 40–70% are attributable to non-shared environmental influences^12,22,23,31^. These findings are typical of those of behavioural genetic studies on individual differences in human traits (including personality traits); shared environmental influences are of minimal significance^32,33^. Although conscientiousness measures are moderately heritable, it is important to note that heritability does not mean immutability^34^. Behavioural genetic studies seek to partition the variance components of variables of interest at a particular time point in a particular sample. Therefore, our results do not imply that the mean scores of conscientiousness measures do not increase or decrease through development or after intervention.

Additionally, we found a high level of genetic correlations among conscientiousness measures, providing further evidence for genetic influence among all four variables; the correlations ranged from 0.82 (0.79–0.84) to 0.92 (0.89–0.93) and individual differences in the propensity to be conscientious stem principally from the same set of hundreds (probably thousands) of variants across the genome (Table 3). Our findings also suggest that non-shared environmental effects play an important role. However, non-shared environmental influences include measurement error, and therefore, may be stochastic rather than systematic. We made little progress in identifying specific non-shared environmental influences, despite the pervasiveness of these influences in relation to a given trait. However, we speculate that differences among person-specific environments produce some degree of individual difference in conscientiousness. For example, a wide range of extra-curricular activities teach students how to behave responsibly and work in teams^35^ and non-clinical mindfulness programs increase the conscientiousness of medical students^36^.

Third, to test formally the idea that conscientiousness measures were genetically coherent, we constructed correlated-factors, independent pathway, and common pathway models; the latter model best fit to the data. This result indicates that all four conscientiousness-related measures can be understood as exemplars of the higher-order common latent factor of conscientiousness. We found that the substantial variance/covariance evident among conscientiousness measures was genetic in nature (Table 3); multivariate genetic analyses showed that the genetic intercorrelations among the four variables of interest were high, and a single set of polygenetic factors explained approximately 70% of the variation in conscientiousness measures (Fig. 2). Moreover, as shown in the bar chart in Fig. 2, over 80% of the genetic influences on each conscientiousness-related measure were derived from the common latent factor (e.g. for conscientiousness, 92% of the genetic influence was due to the common latent factor, whereas 8% was specific). This finding suggests that the broad construct of conscientiousness is highly genetically driven.

Most importantly, our findings demonstrated that a latent common factor substantially explains the covariance among four different measures, and that individual differences in the broad construct of conscientiousness are substantially explained by the genetic influence. Although previous meta-analyses have identified several genetic loci significantly associated with conscientiousness^37,38^, the strong genetic overlap among these measures may facilitate the identification of valid susceptible genetic variants with new methods (e.g. genomic SEM^39^, multi-trait analysis of GWAS [MTAG]^40^).

We obtained moderate non-shared environmental correlations (~ 0.62; Table 3). Of the total variance for the common factor, 28% is explained by non-shared environmental influences (Fig. 2), indicating that covariation between the four conscientiousness-related measures are, to some extent, also due to non-shared environmental influences derived from the common factor. This latent common factor elicits each of the four measures environmentally (and also genetically); as a result, moderate non-shared environmental correlations are reasonable. Approximately 60% of the non-shared environmental influences on each conscientiousness-related measure were derived from the common latent factor (e.g. for conscientiousness, 64% of the non-shared environmental influence was shared, whereas 36% was specific). This finding indicates that, unlike genetic variance, a greater proportion of non-shared environmental variance was due to the unique factor, including measurement error, than to the latent common factor.

Notably, our analyses revealed that conscientiousness-related constructs were both phenotypically and behavioural-genetically similar. Even grit, which was recently listed first among the socio-emotional skills, was highly loaded to the common latent factor of conscientiousness, along with the three other constructs. This finding indicates that grit did not have a greater proportion of variance due to specific genetic and environmental influences. We doubt that these four conscientiousness-related constructs are fully psychologically distinct; the large degree of overlap suggests that they represent a jangle fallacy.

This study has several limitations. First, the research design with twin siblings who are reared together tends to show smaller shared environmental effects than do adoption study design^41,42^, suggesting a downward estimation bias for shared environmental effects. Indeed, a previous meta-analysis found that, for individual differences in personality traits, the AE model provided the best model fit^33^. According to model comparisons in univariate genetic analyses, shown in Table 2, the shared environmental effects are negligible, but not zero, for several variables. Shared environmental influences on adolescent conscientiousness should be carefully discussed when conducting multivariate genetic analyses. Although our final model was an AE common pathway model (Fig. 2), for the purpose of comparison, an ACE common pathway model was also shown in Supplementary Fig. S1, which indicates that downward bias for shared environmental influences was minimal. Second, as we included only Japanese adolescents, our data may not generalise to those of other ages or ethnicities. Among adult and older-aged twins of Western countries, genetic covariations underpinning domains of the Big Five personality traits including conscientiousness were explained by an independent pathway model featuring one or several common genetic factor(s); further study is required^43–46^. Third, our data were cross-sectional in nature; the development of the genetic and environmental architecture of conscientiousness throughout the lifespan remains unclear and requires a longitudinal study. Fourth, the lack of convergence of model selection methods should be mentioned. In terms of BIC, the common pathway model fit best to the data, whereas in terms of the likelihood-ratio chi-square test, all the models fit worse than the baseline model (i.e. correlated factors model), which is another limitation. However, because the BIC performs better with regard to model selection in the context of complex multivariate models^47^, the BIC was preferentially interpreted in this study.

In summary, we found that conscientiousness-related constructs assessed using four different scales strongly correlated both phenotypically and behavioural-genetically, and that the architectures were genetically coherent. Conscientiousness in Japanese adolescents is best conceptualised as a common latent factor with additive genetic and non-shared environmental factors specific for each measure. However, compared to the effects of the common latent factor, the effects of specific genetic and non-shared environmental factors were weaker. Conscientiousness as a latent personality trait factor was genetically accounting for 54–59% of the phenotypic variance, whereas specific additive genetic factors accounted for only 5–10% of the phenotypic variance. This finding indicates that over 84% of the genetic variance was explained by a common genetic factor, suggesting that individual differences within broad conscientiousness constructs are primarily attributable to genetics. Additionally, we found little indication that any shared environmental influence was the source of the phenotypic variance; non-shared environmental influences do exist but exerted smaller effects than the additive genetic influences. However, this does not imply that environmental influences are always irrelevant in terms of the variance/covariance of individual differences in personality traits. Recently, Dick et al.^48^ interestingly suggested that shared and non-shared environmental influences moderate the significance of genetic influences, and that a genetic predisposition can be shaped in part by our environment, such that formal testing of the effects of gene by environmental interactions would be a promising approach for improving our understanding of the developmental and behavioural genetic bases of conscientiousness.

## Methods

### Twin sample

We newly recruited mothers of adolescent twins; we asked them (and the twins) to engage in an online survey run by a Japanese marketing research company. To identify relevant mothers, we initially asked: (a) Are you female? (b) Do you have children? (c) Are your children twins? and, (d) Are your twins aged between 9 and 18 years? If all answers were yes, mothers proceeded to an informed consent screen. Valid informed consent was obtained from all participants prior to administering the online survey. Participants (i.e. twins’ mothers) were compensated with online shopping points after the completion of the online questionnaire. Internet-based survey respondents may engage in careless or inattentive responding. For data cleaning, a marketing research company excluded possible suspected inappropriate responses in terms of the percentage of missing data, too-short completion times, and clearly suspicious response patterns (e.g. the same responses selected for each item of a scale) prior to data delivery.

Valid completed questionnaires were obtained from a total of 1958 families with adolescent twins (590 male MZ twin pairs, 545 female MZ twin pairs, 202 male DZ twin pairs, 209 female DZ twin pairs, and 412 opposite-sex DZ twin pairs). The twins ranged in age from 9 to 18 years (mean = 12.71, standard deviation = 2.73 years), and the mothers from 25 to 68 years (44.71, 5.88). Zygosity was determined by assessing the extent of physical similarity between twins^49^. Both same- and opposite-sex DZ twin pairs were included. As the results barely changed when we removed the opposite-sex DZ twins, we decided to retain them to enhance the statistical power of our work. The study was approved by the institutional ethics committee for experimental psychology research at the Graduate School of Education, Kyoto University, and all research was performed in full accordance with the relevant guidelines and regulations.

### Measures

We measured conscientiousness using four scales: the Chernyshenko Conscientiousness scale (short form) (20 items, e.g. “Rarely jump into something without first thinking about it”)^28^; the effortful control items of the Early Adolescent Temperament Questionnaire Revised (16 items, e.g. “Find it easy to really concentrate on a problem”)^29^; the Brief Self-Control measure (13 items, e.g. “Have a hard time breaking bad habits [reversed item]”)^30^; and the Grit scale (12 items, e.g. “New ideas and new projects sometimes distract me from previous ones [reversed item]”)^8^. Twin mothers were asked to rate their children on a 5-point Likert-type scale ranging from 1 (strongly disagree) to 5 (strongly agree). The Cronbach’s alphas were 0.79 for conscientiousness, 0.86 for effortful control, 0.78 for self-control, and 0.70 for grit; all values were acceptable.

### Statistical analyses

In preliminary phenotypic analyses, we calculated means, standard deviations, correlations, and partial correlations after controlling for twin age and sex, for all four conscientiousness-related scales. Additionally, all four scales were subjected to maximum likelihood factor analysis, using individual twins randomly selected from all twin pairs.

To divide the phenotypic variance of measures of conscientiousness into genetic and environmental contributions, univariate genetic analyses, as described in Neale and Maes^50^, were performed by exploiting the differences in the genetic relatedness of MZ and DZ twin pairs. MZ twins share all genes and the family environment; DZ twins share (on average) half of their genes and the family environment. Given such differences in genetic relatedness, univariate genetic analyses can decompose variance components into additive genetic, non-additive genetic, common (or shared) environmental, and nonshared environmental influences (the latter includes measurement error). The effect of additive genetic factors (A) is assumed to be the sum of contributions from multiple genes that sum to form a quantitative phenotype. If the MZ intraclass correlation is larger than the DZ intraclass correlation, a genetic influence is inferred. If the MZ intraclass correlation is more than double the DZ intraclass correlation, a non-additive genetic (D) influence (assumed to reflect an interactive contribution of alleles within a single locus) is inferred. If the MZ intraclass correlation is less than double the DZ twin correlation, a common environmental (C) influence (not a genetic influence) rendering family members alike is inferred. A nonshared environmental (E) influence reflects differences among family members even when they live together. However, a full ADCE model is not identified if data are available for twins reared together, due to limitations of the model assumptions. If intraclass correlations did not suggest non-additive genetic influences, model fitting results were given for ACE and reduced models (i.e. AE, CE, and E models); similarly if intraclass correlations did not suggest shared environmental influences, model fitting results were given for ADE and reduced models (i.e. AE, and E models). Note that the DE model was not tested because genetic dominance in the absence of genetic additivity is biologically implausible^50^. As we expected that the four variables would be moderately correlated, we next performed multivariate genetic modelling; this decomposes the covariance between variables, and the variance of variables, into genetic and environmental influences. We tested three models: (A) a correlated-factors, (B) an independent pathway, and (C) a common pathway model (Fig. 1).

A correlated-factors model (mathematically equivalent to a Cholesky decomposition solution) extended from a univariate model was initially employed to decompose the covariance between the given traits into genetic and environmental sources of variance, to estimate genetic and environmental correlations. The least restrictive model included A, C (or D), and E effects on each variable, and the extents of all effects were correlated. This model explores the extent to which phenotypic correlations among variables are attributable to correlations between individual, latent genetic and environmental factors.

The second model (the independent pathway model) is more restricted. This model uses a single set of common A, C (or D), and E factors that affect all observed variables directly, and also specific A, C (or D), and E residual factors for all variables. This model explores the extent to which the same genes and similar environments are implicated in covariation among all traits included in the model; common genetic and environmental factors directly influence the observed variables, without an intermediate higher-order factor. As shown in Fig. 1, there is no latent phenotype in the model, only independent genetic and environmental factors. If the independent pathway model provides the best fit with the data, heritability estimates should be calculated for the individual variables because the common genetic and environmental factors do not necessarily cause similar groupings of variables.

The third model tested, the common pathway model, is the most restrictive. This model includes the effects of a common set of A, C (or D), and E factors that influence an underlying phenotypic factor, and measure-specific residual factors influencing each variable. This model explores whether common-factor effects on the latent psychometric factor provide reliable estimates of the genetic and environmental influences on conscientiousness. If the common latent factor is included in the model, each observed variable is an exemplar of that factor. If the common pathway model provides the best fit with the data then the latent phenotype in the model is considered to be a statistically and psychologically valid construct, as it serves to mediate the genetic and environmental covariance among the variables.

When fitting models to raw data, variances, covariances, and means are first freely estimated to obtain a baseline index of fit (i.e. minus twice the log-likelihood; − 2lnL). The − 2lnL under this unrestricted baseline (saturated) model is then subtracted from the − 2lnL under more restrictive biometric models. This result is a likelihood-ratio chi-square test of goodness of fit for the model (*χ*^2^); the *χ*^2^ difference test (Δ*χ*^2^) can also be used as a measure of model fit. However, because log-likelihood ratio testing is problematic in its application to structural equation modelling^51^, and because BIC^26^ has been found to outperform the AIC^52^ calculated for the log-likelihood test and degrees of freedom, in the context of complex multivariate models in larger samples^47^, the BIC was mainly used during model selection in this study.

All behavioural genetic analyses in this study were performed using raw data with the full information maximum likelihood estimation implemented in the R packages *OpenMx* 2.0^53^ and *lavaan*^25^.

## Supplementary Information

Supplementary Information.

## Data Availability

All datasets analysed in the current study are included in this article.
