# Supplementary material for: Genetic and environmental architecture of conscientiousness in adolescence
Source: Sci Rep. 2021 Feb 5;11:3205. doi: 10.1038/s41598-021-82781-5 (PMC7864923; doi:10.1038/s41598-021-82781-5)

Supplementary Information

Genetic and environmental architecture of conscientiousness in adolescence

Yusuke Takahashi[1], Anqing Zheng[2], Shinji Yamagata[3], Juko Ando[4]

1: Graduate School of Education, Kyoto University, Japan; 2: Department of Psychology, University of Illinois at Urbana-Champaign, USA; 3: Graduate School of Education and Human Development, Nagoya University, Japan; 4: Faculty of Letters, Keio University, Japan

Correspondence concerning this article should be addressed to Yusuke Takahashi, Graduate School of Education, Kyoto University, Yoshida Honmachi, Sakyo-ku, Kyoto, 6068501, Japan. Email: takahashi.yusuke.3n@kyoto-u.ac.jp

Supplementary Figure S1 ACE common pathway model for conscientiousness-related measures with standardised estimates (and 95% confidence intervals) alongside bar charts for the percent variance explained.

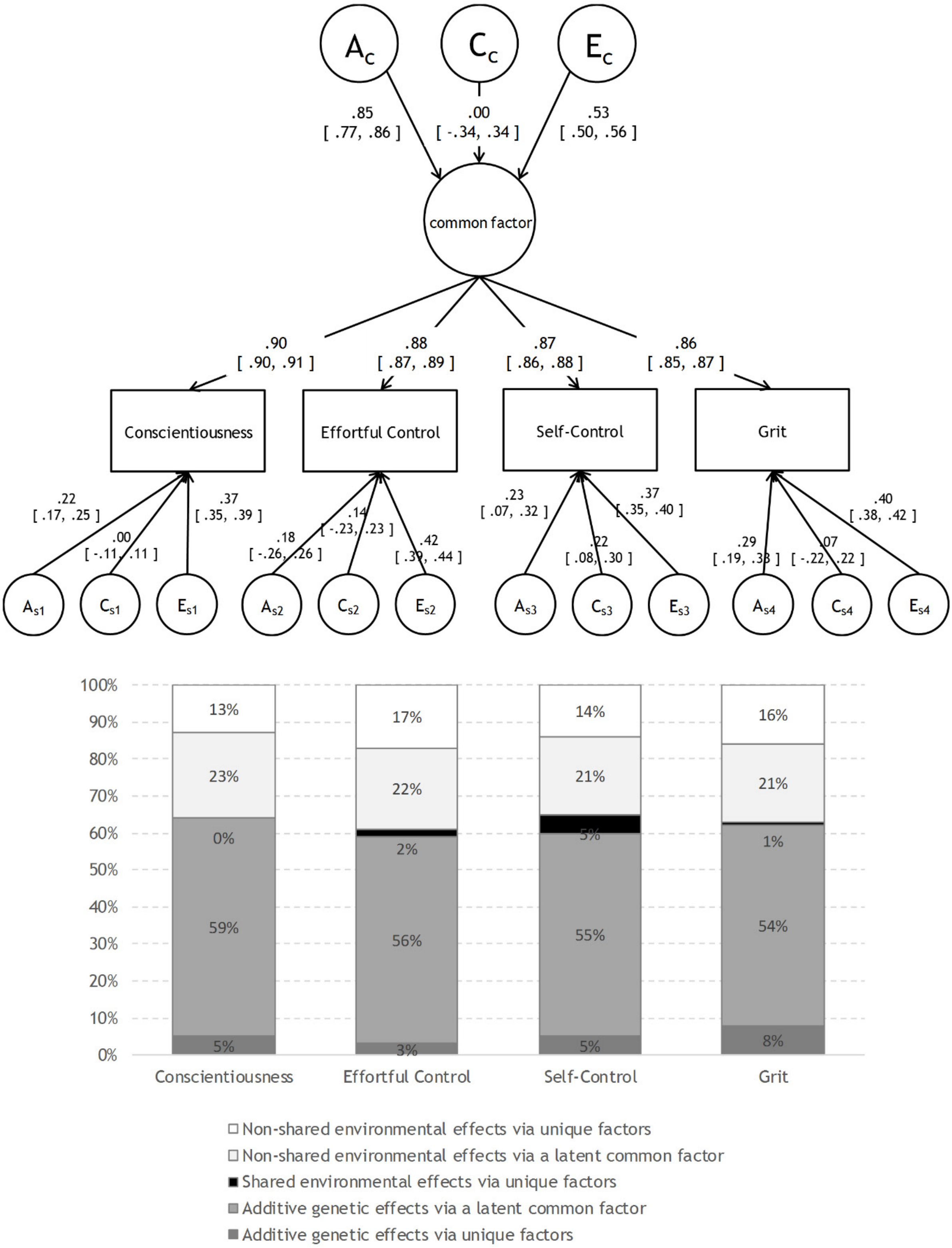

Supplement: Supplementary file 1 — Supplementary Information. [file 41598_2021_82781_MOESM1_ESM.pdf]
